# Boosting recovery before surgery: The impact of prehabilitation on upper gastrointestinal cancer patients – A quantitative comparative analysis

**DOI:** 10.1371/journal.pone.0315734

**Published:** 2025-03-18

**Authors:** Yuping Liu, Xiaoli Chen, Liqun Zou

**Affiliations:** 1 Department of Emergency Medicine, West China Hospital, Sichuan University/West China School of Nursing, Sichuan University, Chengdu, China,; 2 Disaster Medical Center, Sichuan University, Chengdu, China,; 3 Nursing Key Laboratory of Sichuan Province, Chengdu, China; Acibadem Maslak Hospital: Acibadem Maslak Hastanesi, TÜRKIYE

## Abstract

**Background:**

Emerging research presents divergent perspectives on the efficacy of prehabilitation for patients scheduled for surgery due to upper gastrointestinal (GI) cancers, capturing the attention of both the scientific community and surgical professionals. This quantitative comparative analysis seeks to assess the association of prehabilitation to ameliorate postoperative outcomes in individuals with upper GI cancers, thereby providing a comprehensive evaluation of its potential benefits within a surgical context.

**Materials and methods:**

Medline, Embase, Cochrane Library and Web of Science were searched up to February 2024. Studies reporting the association between prehabilitation, and postoperative complications, readmissions or other outcomes of interest were included. Fixed or random effect models were used, and forest plots were applied to show the results of the quantitative comparative analysis.

**Results:**

A total of 198 studies were initially screened based on titles and abstracts, with 129 studies subsequently excluded. Overall, 69 full-text studies were identified, of which 12 studies were finally included for qualitative analysis in the quantitative comparative analysis after determining whether the inclusion and exclusion criteria were met. The pooled results indicated that prehabilitation significantly reduced the overall postoperative complication rates in patients with upper GI cancer undergoing surgical therapy with the pooled OR of 0.59 (95%CI: 0.39–0.88). Moreover, prehabilitation was also shown to be a protective factor of pulmonary complications (OR: 0.54, 95%CI: 0.36–0.80) and ICU readmissions (OR: 0.23, 95%CI: 0.06–0.89).

**Conclusion:**

The correlation between prehabilitation and a reduction in overall postoperative complications, pulmonary complications, and ICU readmissions among patients with upper GI cancer is substantiated by significant data. This pivotal finding necessitates further empirical investigation to validate these initial results and ascertain the clinical efficacy of prehabilitation protocols, thereby informing future surgical practice strategies.

## Introduction

Globally, upper gastrointestinal (GI) cancers rank among the most prevalent cancer types, with 36.4% of cancer-related mortalities in China attributed to these malignancies [1,2]. Esophageal cancer (EC) and gastric cancer (GC) emerge as the predominant forms within this category. Surgical resection stands as a fundamental therapy for the majority of affected individuals [3–5]. However, patients with these cancers often present in a condition of malnutrition and frailty, compounded by a diminished functional reserve—factors intimately linked to adverse postoperative outcomes [6,7]. The investigation to decrease the post-surgical complication rates in upper GI patients presents an ongoing challenge. An important question lingers: Could prehabilitation serve as a pivotal intervention to lower hospital readmission rates and ameliorate the postoperative outcomes in patients with upper GI cancers? The exploration of this possibility is essential for improving surgical success rates in the realm of upper GI oncology.

Prehabilitation emerges as an approach within the preparatory phase, aimed at enhancing the physiological and functional resilience of individuals to better withstand the challenges of major surgery and to support post-surgical recovery [8]. This comprehensive preoperative regimen typically encompassed tailored physical exercise programs, nutritional guidance, and psychological support.

Evidence from prior studies revealed the efficacy of prehabilitation in diminishing postoperative complications among patients who underwent colorectal cancer surgeries [9]. Despite these promising findings, the effectiveness of such interventions specifically for patients with upper GI cancers remains a subject of debate. Therefore, a comprehensive meta-analysis is required to thoroughly investigate the impact of prehabilitation on patients with upper GI cancer and to provide a more definitive understanding of the role that prehabilitation plays in affecting the surgical outcomes for patients with upper GI cancers.

## Materials and methods

The work was conducted in accordance with the “Preferred Reporting Items for Systematic Reviews and Meta-Analyses” (PRISMA statement) [10,11], and assessed the methodological quality of systematic reviews (AMSTAR) Guidelines [12].

### Search design

Medline, Embase, Cochrane Library and Web of Science databases were independently searched, and the search strategy was designed basing on the following keywords: esophageal cancer; gastric cancer; gastroesophageal junction cancer; upper GI cancer and prehabilitation. The search was dated up to February 1, 2024. Reviews, expert opinions, case reports, letters and conference literature were excluded. The details of search strategy were shown in S1 File.

### Study selection

The included studies met the following criteria: (1) patients were histologically or pathologically diagnosed with upper GI cancers and patients had undergone surgery therapy; (2) studies had to report the details and programs of prehabilitation; (3) studies must have included a comparative element, either through randomized controlled trials (RCTs) or cohort studies with a defined control group, which was typically consisted of patients who did not receive the prehabilitation intervention but were managed according to standard care; (4) studies must have reported the odds ratios (ORs) and 95% confidence intervals (95% CIs) of postoperative complications of patients or the associated ORs and 95% CIs could be calculated through the exact data in studies. During the selection process, studies were excluded if: (1) duplicated studies; (2) patients without pathology diagnosis; (3) animal experiments; (4) studies had only utilized nutritional or physiologic support as prehabilitation program without exercise; (5) studies with incomplete data or the exact data could not be retrieved. Two scholars completed a search of databases independently and each was blind to the other. If disagreements occurred, the third scholar was involved to resolve.

### Data extraction

At the time the selected studies were identified and the data extraction was conducted independently. The primary outcomes of interest were overall postoperative complications, postoperative pulmonary complications, and hospital readmission. Overall complications refer to all postoperative complications, encompassing both common and uncommon complications. Data were additionally collected including some uncommon postoperative complications such as anastomotic leakage, cardiac complications, wound infections, vocal cord palsy, chylothorax and postoperative bleeding. Associated ORs of relevant outcomes with their 95% CIs were retrieved or calculated from the reported results. And the other data necessary for this meta-analysis include the first author’s name, publication date, study design, surgery type, sample size, study population, therapeutic methods, cancer types, tumor stages, the exact programs of prehabilitation et al. were also retrieved from the published studies.

### Quality assessment

In this meta-analysis, any cumulative bias may cause deviation in evaluating the effects of prehabilitation. Quality assessment of the incorporated studies was evaluated basing on the Newcastle–Ottawa Quality Assessment Scale (NOS) and Cochrane Collaboration’s Risk of Bias Tool to achieve the study quality assessment based on different study designs. The NOS was adopted for nonrandomized controlled studies, and studies with more than 6 scores were considered to a high quality. Cochrane Collaboration’s Risk of Bias Tool was utilized to evaluate the risk of bias in RCT.

### Statistical analysis

Forest plots were used to visualize the effect size and results of included studies in this meta-analysis. The heterogeneity among included studies was tested through I^2^ statistics. If the I^2^ value was greater than 50%, the heterogeneity among studies was significant, and the random-effects model was used to analyze. Conversely, when the I^2^ value was lower than 50%, the heterogeneity was moderate, and the fixed-effects model was applied.

When more than 5 studies were included for any outcome of this meta-analysis, sensitivity analysis was conducted through leave-one-out method to determine the reliability of the pooled effects size in the meta-analysis. Further, Egger’s test was applied through the P value and asymmetry of funnel plot to evaluate the potential publication bias among included studies. Analysis of the data in this meta-analysis was conducted by utilizing the Review Manager 5.4 software package.

## Results

### Search results

**Fig 1:**
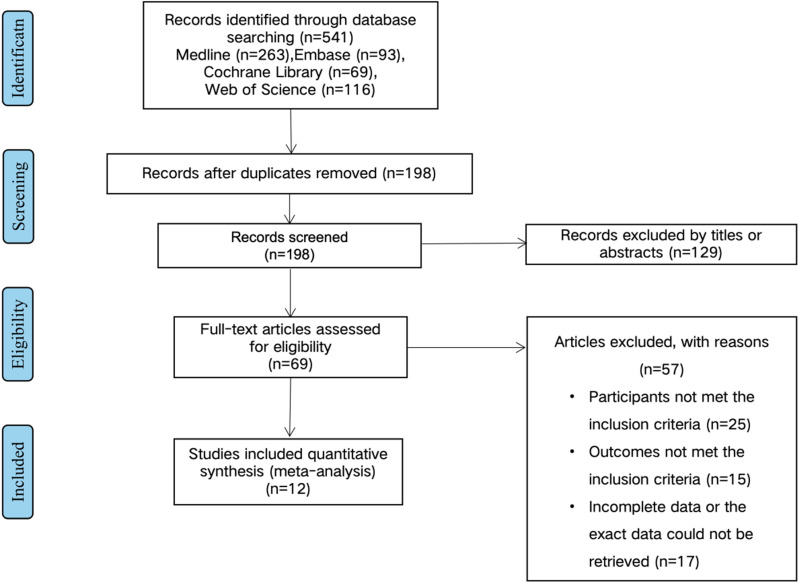
The flow diagram showing the process of study selection.

The selection of included studies is shown in the flow chart (Fig 1). A total of 541 studies were found based on the initial search strategy. After duplicates were removed, 343 studies were excluded. By reviewing the titles and abstracts of the remaining studies, 129 studies were further excluded. The last 69 relevant studies underwent full-text screening; 57 studies were then excluded because of non-conformity with the inclusion criteria or incomplete data. The detail reasons and excluded research were listed in S2 Table. Ultimately, 12 studies were included in the meta-analysis.

### Study characteristics

The characteristics of all included studies are shown in Table 1. The total number of included patients was 832. The sample size in each study ranged from 48 to 111 patients. Over half of the included patients were from Western countries. In addition, the program of prehabilitation varied in different studies. 4 studies used exercise programs, personalized nutrition support and psychological counseling as the preoperative prehabilitation; patients in 3 studies received exercise therapy combined with individual nutritional support. In 5 studies, patients solely received exercise including aerobic exercise, resistance exercise and inspiratory muscle training (IMT) et al. All included patients underwent neoadjuvant therapy and/or surgical therapy. Among all the included patients, 509 patients underwent neoadjuvant therapy combined with surgery, and 323 patients only received surgical resection. The details of prehabilitation can be found in S1 Table.

**Table 1:** The characteristics of the included studies.

Name	Year	Design	Treatment	Surgery type	Population	Cancer type	Pretreatment Intervention	N	Male	Female	Stage	N IG	N CG	Age		NOS score Risk of bias
														IG	CG	
Laura [13]	2023	RCS	NCR + S	MIE + Open esophagectomy	UK	EC	E + N + P	79	59	20	I–IV	51	28	66.2 ± 9.9	63.5 ± 9.6	7
Thijs [14]	2022	RCS	NCR + S	MIE-IL	UK	EC	E + N + P	95	74	21	0–IV	52	43	64 ± 8	65 ± 9	7
Sophie [15]	2022	RCT	NCR + S	Open esophagectomy + Total gastrectomy	UK	EC+GC	E + N + P	48	NR	NR	I–IV	24	24	65 ± 6	62 ± 9	^*^
Yuma [16]	2022	RCS	S	Distal gastrectomy + Total gastrectomy	Japan	GC	E + N	58	40	18	I–IV	15	43	74.9 ± 2.5	70.7 ± 1.7	7
Laura [17]	2020	PCS	NAC + S	Open esophagectomy	UK	EC	E + N + P	111	NR	NR	I–IV	72	39	61-74		8
Yuji [18]	2020	RCS	NAC + S	MIE + Open esophagectomy	Japan	ESCC	E	48	38	10	I–IV	23	25	65.9 ± 7.7	65.6 ± 8.7	7
Christensen [19]	2018	PCS	NCR + S	MIE-IL + RAMIE + Open esophagectomy	Denmark	GOJ	E	50	45	5	I–III	21	29	Mean: 64.8		8
Enrico [20]	2018	RCT	NCR + S	MIS + Open surgery	Canada	EC+GC	E + N	51	38	13	I–III	26	25	67.3 ± 7.4	68.0 ± 11.6	^*^
Yuji [21]	2017	RCS	NCR + S	MIE + Open esophagectomy	Japan	EC	E	52	41	11	0–IV	31	21	64.2 ± 8.9	64.9 ± 9.1	7
Kazuyoshi [22]	2016	Prospective non-RCT	S	MIG + Open gastrectomy	Japan	GC	E + N	90	52	37	I–IV	22	68	75 ± 5	72 ± 4	8
Haruhiko [23]	2014	Prospective non-RCT	S	MIG + Open gastrectomy	Japan	GC	E	72	69	3	I–III	18	54	63.1 (51-76)	66.1 (39-81)	8
Daniela [24]	2012	Pragmatic non-RCT	NCR + S	MIE + Open esophagectomy	Netherland	EC	E	78	58	20	NR	39	39	65.4 ± 7.5	66.5 ± 9.6	8

* The detailed results of Cochrane Collaboration’s Risk of Bias of two RCTs included in this meta-analysis were shown in S1 Figure.

RCS, retrospective cohort studies; PCS, prospective cohort studies; RCT, randomized controlled trial; NCR, neoadjuvant chemoradiotherapy; NAC, neoadjuvant chemotherapy; S, surgery; MIE, minimally invasive esophagectomy; MIE-IL, minimally invasive Ivor-Lewis esophagectomy; RAMIE, robot-assisted minimally invasive esophagectomy; MIS, minimally invasive surgery; MIG, minimally invasive gastrectomy; EC, esophageal cancer; ESCC, esophageal squamous cell cancer; GOJ, gastroesophageal junction cancer; GC, gastric cancer; E, exercise; N, nutritional support; P, physiologic support; IG, intervention group; CG, control group; NOS, Newcastle–Ottawa Quality Assessment Scale.

### Quality assessment

Based on different study designs, the results of NOS and Cochrane Collaboration’s Risk of Bias Tool were demonstrated in Table 1 and the included studies were shown to have high quality. The detailed results of Cochrane Collaboration’s Risk of Bias of two RCTs included in this meta-analysis were shown in S1 Fig. The specifics of the NOS score are provided in S3 Table.

### Association of prehabilitation with postoperative complications

**Fig 2:**
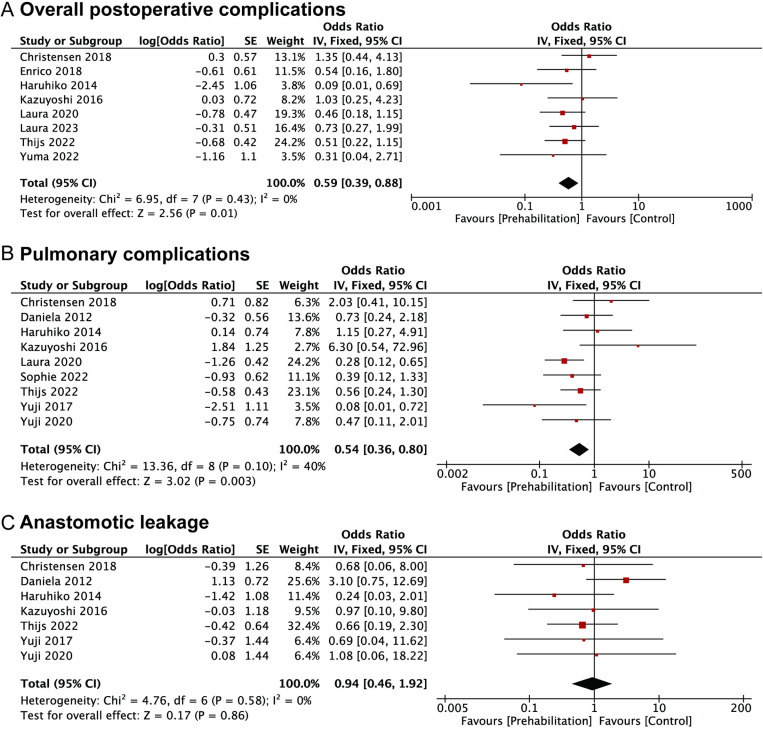
Forest plots of studies evaluating ORs of prehabilitation and postoperative outcomes of upper GI cancers, stratified by (A) overall postoperative complications; (B) pulmonary complications; (C) anastomotic leakage.

The results of the meta-analysis showed that prehabilitation had a positive effect on certain postoperative outcomes in upper GI cancer patients. All data extracted from the primary research relevant to this study have been provided in S4 Table. Specifically, patients who underwent prehabilitation had lower overall postoperative complication rates, with a pooled OR of 0.59 (95%CI: 0.39–0.88) (Fig 2A). Based on the value of I^2^ (I^2^ = 0.0%), we considered that the heterogeneity was low, and the fixed-effects model was applied.

9 studies investigated the correlation between prehabilitation and postoperative pulmonary complications. The results presented that the incidence of postoperative pulmonary complication in the prehabilitation groups was lower than the control groups, with a pooled OR of 0.54 (95%CI: 0.36–0.80) (Fig 2B). A fixed-effects model was used to evaluate heterogeneity, and the value of I^2^ was 40%.

In this meta-analysis, 7 studies reported an association between prehabilitation and anastomotic leakage which was a severe complication after surgical resection. According to the value of I^2^ (I^2^ = 0.0%), we considered the heterogeneity was low, and the fixed-effects model was used. The pooled OR of anastomotic leakage was 0.94 (95%CI: 0.46–1.92), and no statistical association was found (Fig 2C).

**Fig 3:**
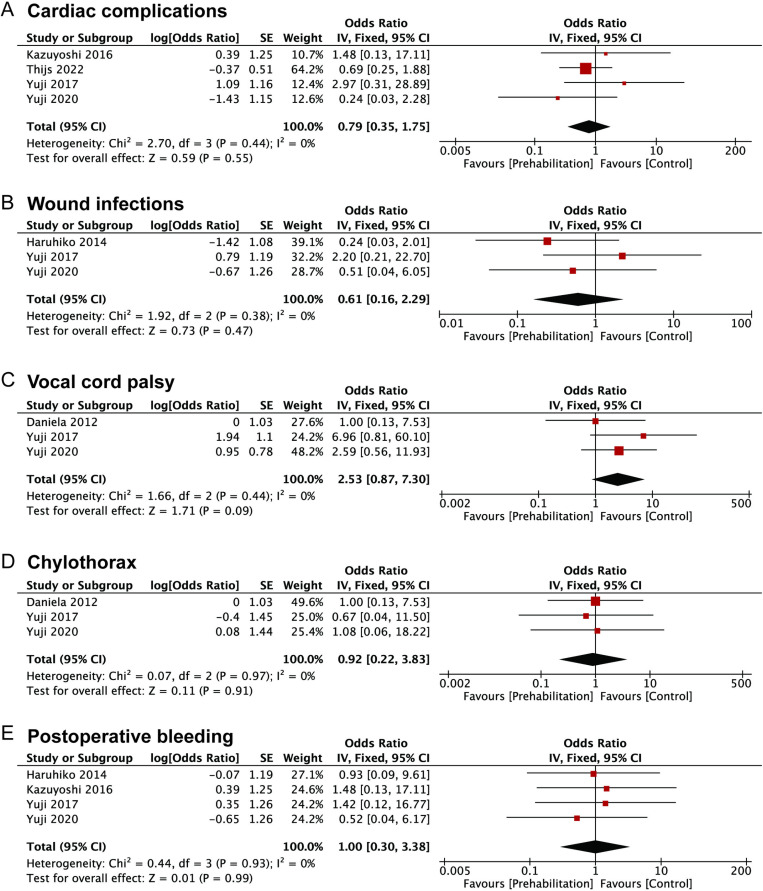
Forest plots of studies evaluating ORs of prehabilitation and (A) cardiac complications (B) wound infections (C) vocal cord palsy (D) chylothorax and (E) postoperative bleeding of upper GI cancers.

In addition, we investigated the impact of prehabilitation on other uncommon postoperative complications in patients with upper GI cancers, including cardiac complications (OR: 0.79, 95%CI: 0.35–1.75), wound infections (OR: 0.61, 95%CI: 0.16–2.29), vocal cord palsy (OR:2.53, 95%CI: 0.87–7.30), chylothorax (OR: 0.92, 95%CI: 0.22–3.83) and postoperative bleeding (OR: 1.00, 95%CI: 0.30–3.38). However, statistical significance was not detected, for the 95%CI crossed with the null line (Fig 3A–E).

### Association of prehabilitation with readmission

**Fig 4:**
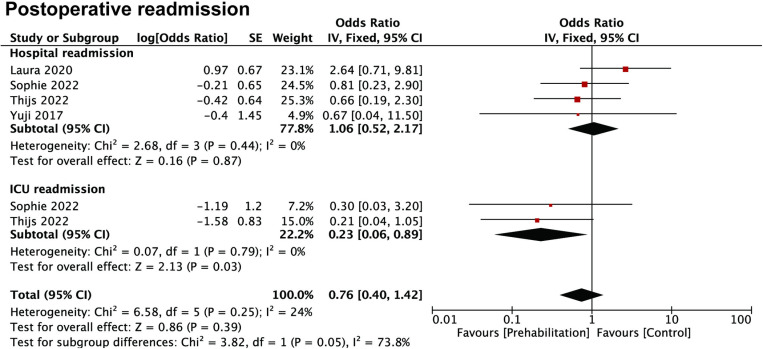
Forest plots of studies evaluating ORs of prehabilitation and postoperative readmission.

Postoperative complications are common problems after surgery and may result in patients requiring additional treatment or recapitalization. In some severe cases, patients need to be readmitted to the intensive care unit (ICU) of a hospital to ensure their recovery and rehabilitation. Therefore, we also explored whether preoperative prehabilitation could reduce the risk of patient readmission. In all of 6 studies that reported the OR of prehabilitation hospital readmission in patients with upper GI cancers, the results showed that prehabilitation might be a positive factor in reducing ICU readmission (OR: 0.23, 95%CI: 0.06–0.89) but not normal hospital readmission (OR: 1.06, 95%CI: 0.52–2.17) (Fig 4).

### Subgroup analyses

**Fig 5:**
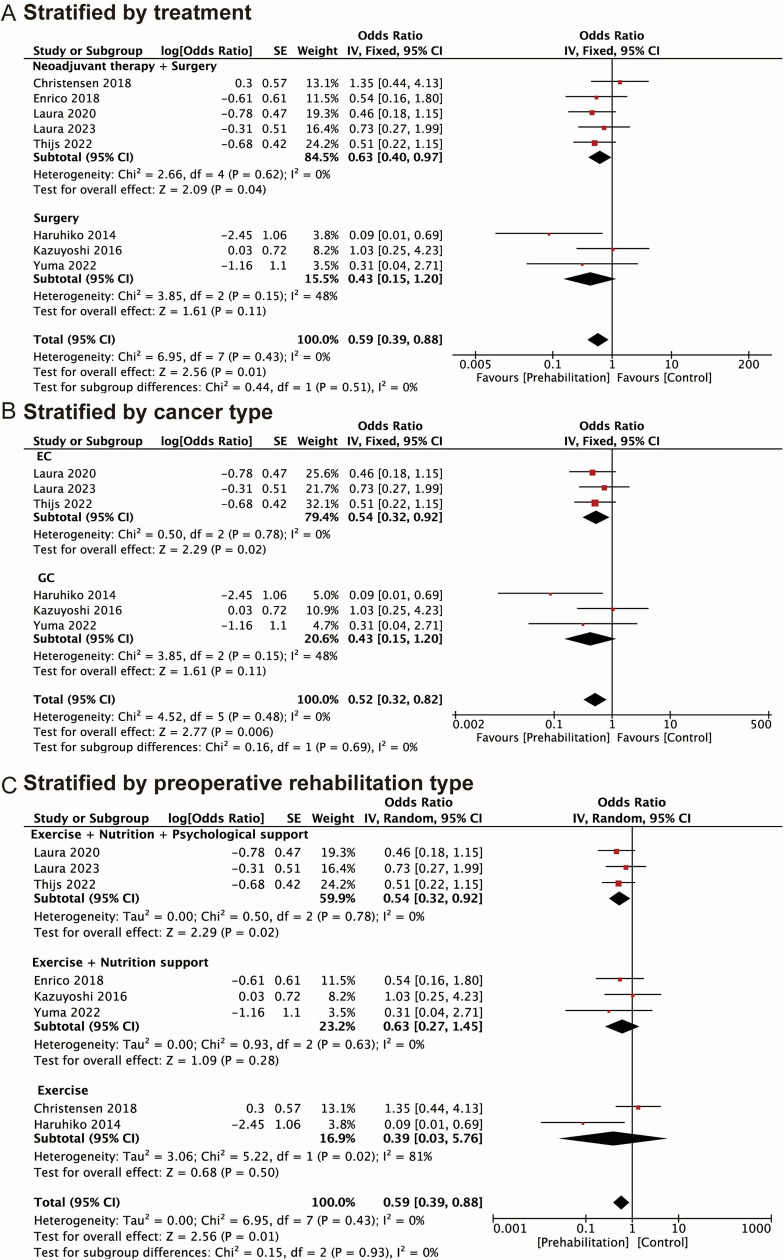
Forest plots of ORs for overall postoperative complications in prehabilitation patients compared with control groups stratified by (A) treatment; (B) cancer type; (C) prehabilitation type. OR: odds ratio; EC: esophageal cancer; GC: gastric cancer.

We conducted subgroup analyses to delve into the protective effect of prehabilitation and to elucidate potential risk factors for postoperative complications among upper GI cancer patients, based on various clinical characteristics outlined in the included studies. Subgroup analysis, stratified by different treatments, revealed a significant correlation between prehabilitation and reduced rates of postoperative complications (OR: 0.63, 95% CI: 0.40–0.97) among patients who underwent neoadjuvant therapy combined with surgical resection. However, such correlation was not evident among patients who underwent surgical therapy alone (OR: 0.43, 95% CI: 0.15–1.20) (Fig 5A). When classified by different cancer types, prehabilitation emerged as a positive indicator in EC patients (OR: 0.54, 95% CI: 0.32–0.92), but no statistical significance was detected among GC patients (OR: 0.43, 95% CI: 0.15–1.20) (Fig 5B). Furthermore, we conducted subgroup analyses to investigate the impact of different types of prehabilitation on postoperative complications. The results indicated that patients who received a personalized exercise program along with nutritional and psychological support had a lower overall rate of postoperative complications, with a pooled OR of 0.54 (95% CI: 0.32–0.92). However, for patients who received exercise alone (OR: 0.39, 95% CI: 0.03–5.76) or exercise combined with nutritional support (OR: 0.63, 95% CI: 0.27–1.45), such correlation was not statistically significant (Fig 5C).

**Fig 6:**
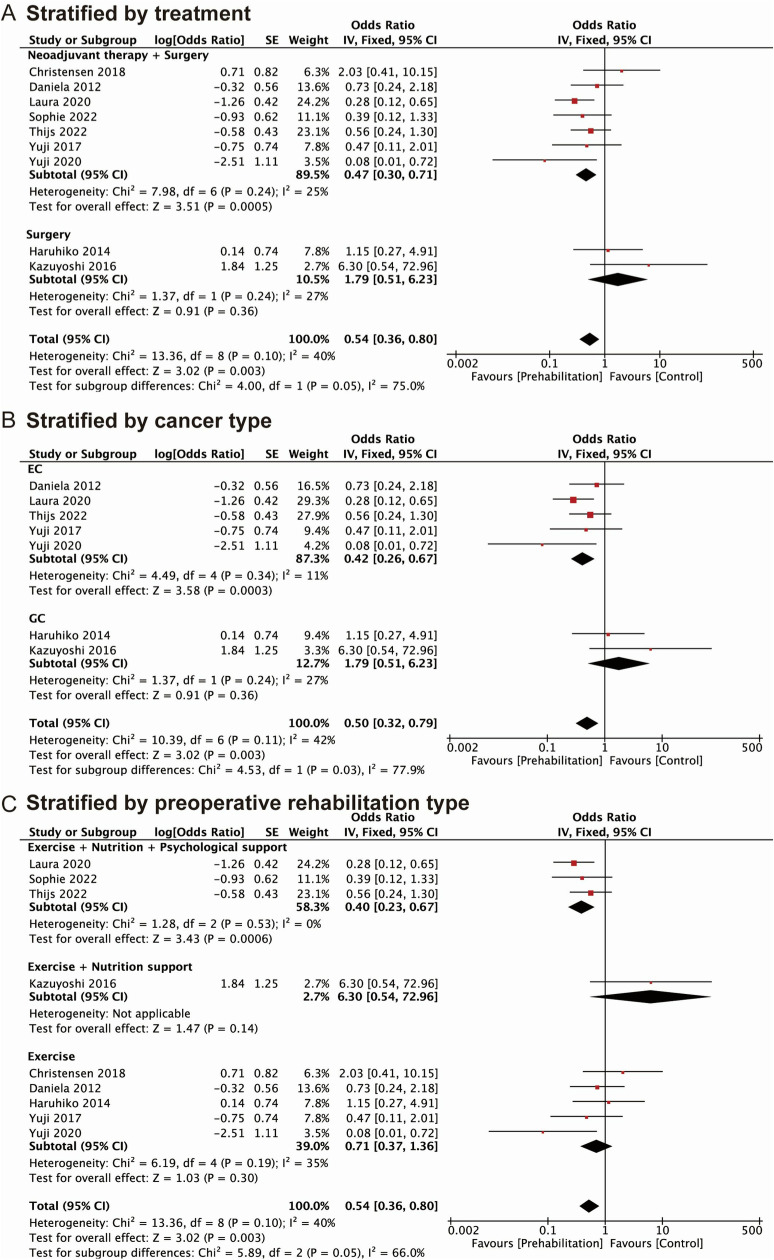
Forest plots of ORs for postoperative pulmonary complications in prehabilitation patients compared with control groups stratified by (A) treatment; (B) cancer type; (C) prehabilitation type. OR: odds ratio; EC: esophageal cancer; GC: gastric cancer.

Furthermore, subgroup analysis was conducted to assess the impact of prehabilitation on postoperative pulmonary complications. Subgroup analysis by treatment methods revealed that prehabilitation served as a protective factor against postoperative pulmonary complications in upper GI patients who underwent neoadjuvant therapy combined with surgery (OR: 0.47, 95% CI: 0.30–0.71). However, no statistical significance was detected in patients receiving surgical treatment alone (OR: 1.79, 95% CI: 0.51–6.23) (Fig 6A). Upon analyzing subgroups based on different cancer types, we demonstrated that prehabilitation significantly reduced the postoperative pulmonary complications rate in patients with EC (OR: 0.42, 95% CI: 0.26–0.67) but not in GC patients (OR: 1.79, 95% CI: 0.51–6.23) (Fig 6B). When stratified by the program of prehabilitation, exercise combined with nutritional and psychological support proved to be an effective prehabilitation method in decreasing the rate of postoperative pulmonary complications (OR: 0.40, 95% CI: 0.23–0.67). However, such correlation was not observed in patients receiving exercise alone (OR: 0.71, 95% CI: 0.37–1.36) or exercise combined with nutritional support (OR: 6.30, 95% CI: 0.54–72.96) (Fig 6C).

### Sensitivity analysis and publication bias

**Fig 7:**
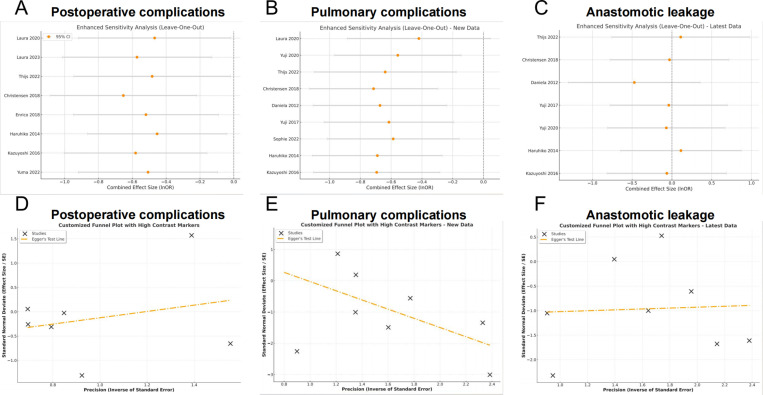
Sensitivity analysis for meta-analysis of prehabilitation for (A) overall postoperative complications; (B) pulmonary complications; (C) anastomotic leakage. Funnel plots of publication bias for meta-analysis of prehabilitation for (D) overall postoperative complications; (E) pulmonary complications; (F) anastomotic leakage.

To evaluate the reliability and stability of the original analysis, a sensitivity analysis was performed by sequentially removing each study. The sensitivity analysis showed that the postoperative complications of the original analysis were not affected by removing any single study (Fig 7A–C). In addition, Egger’s test was used to test for any hidden publication bias. A symmetrical appearance was detected in the funnel plot (Fig 7D–E). The P values of Egger’s test were 0.40, 0.30 and 0.53 for OR of overall postoperative complication, pulmonary complication, and anastomotic leakage respectively. Thus, no significant publication bias was detected.

## Discussion

Prehabilitation represents a significant development in healthcare, focusing on improving patient readiness for surgical and oncological interventions [20]. This proactive approach diverges from traditional rehabilitation by focusing on enhancing both physical and psychological resilience prior to medical procedures. Tailored to individual needs, prehabilitation programs integrate exercise regimens, nutritional counseling, and psychological health support to enhance patients against the injury during the whole period of surgery and treatment [25]. Highlighting the specific benefits for upper GI cancer patients, recent research, including a PRISMA-aligned meta-analysis by Lambert et al. [26], underscores the potential of prehabilitation to elevate functional capacity and curtail postoperative complications. However, their meta-analysis was across a spectrum of cancer types, including hepatobiliary, colorectal, and upper GI cancers, which was constrained to 3 studies focusing on upper GI cancer patients, highlighting the scarcity of comprehensive data in this area [26]. Moreover, a previous study conducted by Sarah et al. included patients with all kinds of GI cancer, encompassing both benign and malignant tumors, which contributes to heterogeneity in patient populations [27]. With a comprehensive review of 12 studies specifically examining the role of prehabilitation in upper GI cancers, this analysis not only reaffirms the positive outcomes associated with prehabilitation but also extends its findings to cover a range of postoperative complications and hospital readmission rates. Through detailed subgroup analyses by cancer types and treatment modalities, this work offers a nuanced understanding of prehabilitation’s role in optimizing surgical outcomes, presenting a compelling case for its broader application in clinical practice.

Our research highlights a clear advantage of prehabilitation over conventional care models for surgical patients, particularly by demonstrating its efficacy in reducing postoperative complications. The most significant impact of prehabilitation appears in the reduction of pulmonary complications, which remain a leading cause of postoperative morbidity and mortality. Pulmonary complications, including pneumonia, respiratory distress syndrome, and atelectasis, are common after upper gastrointestinal (GI) surgeries due to prolonged anesthesia, impaired diaphragmatic function, and immobility. Prehabilitation, through tailored respiratory exercises, physical conditioning, and nutritional optimization, helps improve respiratory mechanics and functional capacity, thereby reducing the incidence of these complications. Our findings underscore this benefit, with a marked decrease in pulmonary complications observed across the studies analyzed. However, the benefit of prehabilitation does not extend uniformly across all types of postoperative complications. In our review of 7 studies specifically assessing anastomotic leakage rates in upper GI cancer patients, prehabilitation showed no significant effect on reducing the incidence of this serious complication. Anastomotic leakage, often caused by impaired healing of the surgical join between sections of the gastrointestinal tract, remains one of the most feared postoperative complications due to its association with high rates of sepsis, reoperations, and mortality. The lack of impact from prehabilitation in this area suggests that factors like intraoperative technique, tissue perfusion, and local infection control play a more critical role in mitigating anastomotic leakage than preoperative conditioning alone. Nutritional status, immune competence, and the local inflammatory response may be more influential in this regard, and interventions focused on enhancing surgical precision or improving postoperative monitoring may be necessary to address this complication. In addition to pulmonary outcomes, our analysis identified other areas where prehabilitation might contribute to postoperative recovery. While prehabilitation did not significantly reduce overall hospital readmission rates, it was associated with a notable decrease in ICU readmissions, indicating that patients undergoing prehabilitation may have fewer severe postoperative complications necessitating intensive care. This finding suggests that prehabilitation improves not only immediate recovery but also the stability of patients during the early postoperative period, potentially due to enhanced cardiovascular resilience and metabolic recovery. Although reductions in cardiac complications, wound infections, and chylothorax were observed in some studies, these trends did not reach statistical significance, likely due to the limited sample sizes or the multifactorial nature of these complications. Cardiac complications, for instance, may be influenced by pre-existing comorbidities such as hypertension and diabetes, which might not be fully mitigated by prehabilitation interventions alone. Overall, while our study shows encouraging results for the role of prehabilitation in reducing certain postoperative complications, such as pulmonary and ICU readmissions, the limited effect on anastomotic leakage and other surgical-specific complications calls for further investigation.

Our investigation unveiled a compelling correlation between prehabilitation and ameliorated postoperative outcomes among patients who underwent a combined regimen of neoadjuvant therapy and surgical resection. Intriguingly, this advantageous association was not evident in patients who underwent surgery alone, implying a distinct advantage for those receiving neoadjuvant therapy in conjunction with prehabilitation efforts. This suggests that patients who underwent neoadjuvant therapy may indeed benefit from having a longer preoperative period, allowing more time for prehabilitation to be effective. This extended timeframe could facilitate greater improvements in physical conditioning, nutritional status, and psychological well-being, thereby contributing to better postoperative outcomes compared to patients who had upfront surgery. Our subgroup analysis further refines these insights, pinpointing a significant association between prehabilitation and superior postoperative outcomes specifically in EC patients, whereas such benefits were not conclusively observed in GC patients. One possible explanation for this discrepancy is that EC surgeries, particularly esophagectomies, are more extensive and have higher associated postoperative morbidity, which might make patients more susceptible to the benefits of prehabilitation in terms of improving cardiorespiratory fitness and overall physical condition. In contrast, GC, while complex, may not present the same level of postoperative complications where prehabilitation could exert a pronounced effect. Based on the results of subgroup analysis, patients may benefited more in prehabilitation group which combined exercise, nutritional and psychological support. That might be partly explained by the reason that exercise helps improve physical conditioning [27], but without adequate nutritional support, patients may lack the necessary resources to fully benefit from exercise. Nutritional support ensures that the body has sufficient energy, proteins, and micronutrients to promote tissue repair, immune function, and muscle strength, thus enhancing recovery [28]. In addition, psychological support is also crucial in helping patients manage the anxiety and stress associated with cancer and major surgery. Addressing psychological well-being can improve adherence to both exercise and nutritional interventions, while also reducing perioperative stress responses, which may negatively impact immune function and recovery [29]. Integrating psychological support into prehabilitation programs helps to optimize overall patient engagement and resilience, contributing to better surgical outcomes. Therefore, the combination of exercise, nutrition, and psychological support provides a more holistic approach to prehabilitation, addressing not only the physical but also the metabolic and emotional needs of the patient.

Our research highlights prehabilitation as a promising strategy that has the potential to improve the treatment outcomes for patients with upper GI cancers. Considering these compelling benefits by the meta-analysis, prehabilitation warrants recognition as a cornerstone of standard care for individuals diagnosed with upper GI cancers. It is imperative for healthcare practitioners to champion the integration of comprehensive prehabilitation programs within existing treatment paradigms, heralding a new era of enhanced patient support and improved clinical outcomes.

Our comprehensive meta-analysis has synthesized all accessible evidence to illuminate the role of prehabilitation as a safeguard for patients grappling with upper GI cancer. However, this exploration is not without its constraints. Firstly, the baseline characteristics of the intervention and control groups varied across the included studies. For instance, while most studies matched for age, sex, and cancer stage, some differences in nutritional status, physical conditioning, and preoperative health were present. These baseline differences could have confounded the reported associations between prehabilitation and postoperative outcomes, particularly in studies where such variables were not adequately controlled. For example, patients with better baseline physical fitness or nutritional status may have been more likely to be placed in the prehabilitation group or to benefit more from prehabilitation, thereby inflating the apparent effectiveness of the intervention. In addition to the variation in baseline characteristics between intervention and control groups, other sources of bias were identified. Selection bias may have played a role in all 10 retrospective cohort studies, where patients selected for prehabilitation may have been those with fewer comorbidities or better general health, making them more likely to have favorable postoperative outcomes regardless of the intervention. Furthermore, publication bias is always a concern in meta-analyses, particularly given the relatively small number of studies included in certain subgroups. Although no significant publication bias was detected via funnel plot and Egger’s test, the small sample sizes in some studies may limit the robustness of these analyses. Moreover, A notable shortfall lies in the diversity in prehabilitation programs—spanning the spectrum of duration, exercise types, and nutritional strategies—which introduces variability that challenges the establishment of concrete conclusions. The absence of a standardized prehabilitation regimen further complicates the interpretation of our results. Another limitation of this meta-analysis is the number of studies included in some of our subgroup analyses is relatively small which might limit the statistical power and may not allow for definitive conclusions or strong hypothesis generation. As a result, the conclusions drawn from them should be interpreted with caution. Moreover, the enduring question of prehabilitation’s influence on long-term postoperative survival remains unanswered, as existing studies fall short of shedding light on these critical outcomes. Therefore, further clinical research is necessary.

## Conclusions

Integrating resistance training, aerobic exercises, respiratory muscle strengthening, along with personalized nutritional and psychological support, prehabilitation shows potential in enhancing postoperative recovery for surgical patients. However, given the significant variability in prehabilitation programs, as well as the small number of patients and limited RCTs, our findings should be interpreted with caution. While prehabilitation may offer benefits in optimizing patients’ physiological states and aiding recovery, further large-scale, standardized trials are necessary to confirm its effectiveness and determine how best to integrate it into the standard care protocol for upper GI cancers.

## Supporting information

S1 Fig The detailed results of Cochrane Collaboration’s Risk of Bias of two RCTs. (PDF)

S1 Table The details of the prehabilitation regimen. (DOCX)

S1 File The details of search strategy. (DOCX)

S2 Table Reasons for exclusion of the excluded studies. (XLSX)

S3 Table The specifics of the NOS score of observation studies. (XLSX)

S4 Table Data extracted from the primary research relevant to this study. (XLSX)

PRISMA 2020 Checklist The PRISMA 2020 statement: an updated guideline for reporting systematic reviews. (PDF)

## Abbreviations

GI: Gastrointestinal
OR: Odds Ratio
CI: Confidence Interval
ICU: Intensive Care Unit
PRISMA: Preferred Reporting Items for Systematic Reviews and Meta-Analyses
AMSTAR: Assessing the Methodological Quality of Systematic Reviews
NOS: Newcastle–Ottawa Quality Assessment Scale
RCT: Randomized Controlled Trial
NCR: Neoadjuvant Chemoradiotherapy
NAC: Neoadjuvant Chemotherapy
S: Surgery
MIE: Minimally Invasive Esophagectomy
MIG: Minimally Invasive Gastrectomy
IMT: Inspiratory Muscle Training
EC: Esophageal Cancer
GC: Gastric Cancer
E: Exercise
N: Nutritional Support
P: Psychological Support
IG: Intervention Group
CG: Control Group
PCS: Prospective Cohort Studies
RCS: Retrospective Cohort Studies
RAMIE: Robot-Assisted Minimally Invasive Esophagectomy
GOJ: Gastroesophageal Junction Cancer.
