# Supplementary material for: Boosting recovery before surgery: The impact of prehabilitation on upper gastrointestinal cancer patients – A quantitative comparative analysis
Source: PLoS One. 2025 Mar 18;20(3):e0315734. doi: 10.1371/journal.pone.0315734 (PMC11918424; doi:10.1371/journal.pone.0315734)
Supplement: S1 Fig — (PDF) [file pone.0315734.s001.pdf]

	Random sequence generation (selection bias)	Allocation concealment (selection bias)	Blinding of participants and personnel (performance bias)	Blinding of outcome assessment (detection bias)	Incomplete outcome data (attrition bias)	Selective reporting (reporting bias)	Other bias
Enrico 2018	+	?	+	+	+	+	?
Sophie 2022	+	?	?	+	+	+	?
