# Supplementary material for: Boosting recovery before surgery: The impact of prehabilitation on upper gastrointestinal cancer patients – A quantitative comparative analysis
Source: PLoS One. 2025 Mar 18;20(3):e0315734. doi: 10.1371/journal.pone.0315734 (PMC11918424; doi:10.1371/journal.pone.0315734)
Supplement: S1 Table — (DOCX) [file pone.0315734.s002.docx]

**Table S1**

| **Author** | **Year** | **Design** | **Surgery type** | **Neoadjuvant therapy** | **Population** | **Cancer type** | **Treatment** | **Pretreatment Intervention** | **N** | **Male** | **Female** | **Stage** | **N Prehabilitation** | **N Control** | **Age** | | **NOS score** | **Duration** | **Intervention** | **Control** |
| --- | --- | --- | --- | --- | --- | --- | --- | --- | --- | --- | --- | --- | --- | --- | --- | --- | --- | --- | --- | --- |
|  |  |  |  |  |  |  |  |  |  |  |  |  |  |  | **Preheabilitation group** | **CC group** |  |  |  |  |
| Laura et al. | 2023 | Retrospective cohort study | MIE + Open esophagectomy | Overall | UK | EC | NCR+surgery | Exercise + Nutrition + Psychological support | 79 | 59 | 20 | I-IV | 51 | 28 | 66.2 ± 9.9 | 63.5 ± 9.6 | 7 | About preoperative 16 weeks | PREPARE program mainly including: Exercise: According to the WHO guidelines, patients were prescribed a personalized exercise program with a defined frequency, intensity, and durationby a experienced exercise therapist Nutritional support: A specialist nutritionist evaluated the nutritional status of patients. Personalised diet plan was designed based on physical condition of each patients Psychological support: Psychotherapies were applied to control anxiety, facilitate adaptation to patients' current psychological health and disease state | Standard-care control group |
| Thijs et al. | 2022 | Retrospective cohort study | MIE-IL | Overall | UK | EC | NCR+surgery | Exercise + Nutrition + Psychological support | 95 | 74 | 21 | 0-IV | 52 | 43 | 64 ± 8 | 65 ± 9 | 7 | About preoperative 13 weeks | PREPARE program mainly including: Personalized nutritional and physical goals were set and adjusted throughout the program. Patients were contacted by the physiotherapists each week to evaluate a weekly training schedule Mental state and psychological health were discussed during visits to the outpatient clinic | Standard-care control group |
| Sophie et al. | 2022 | RCT | Open esophagectomy + Total gastrectomy | Overall | UK | EC+GC | NCR+surgery | Exercise + Nutrition + Psychological support | 48 | NR | NR | I-IV | 24 | 24 | 65 ± 6 | 62 ± 9 | * | About preoperative 15 weeks | Exercise scientist with expertise in cancer care, for 1 h, twice weekly for 15 preoperative weeks, psychological intervention and needs-based nutritional interven tions with frequent, tailored, dietetic input from specialist dieticians | Standard esophagogastric care pathway |
| Yuma et al. | 2022 | Retrospective cohort study | Distal gastrectomy + Total gastrectomy | \ | Japan | GC | Surgery | Exercise + Nutrition support | 58 | 40 | 18 | I-IV | 15 | 43 | 74.9 ± 2.5 | 70.7 ± 1.7 | 7 | Median: 13 days (7-22) | Exercise: walking was recommended about 1 h per day and patients were asked to perform leg press, leg lunge, and squats according to normal activities of daily life of each patient by rehabilitation specialists for resistance training Nutritional support: a total daily caloric intake of 25–30 kcal/kg ideal body weight (IBW) and a daily protein intake of 0.8–1.2 g/kg IBW | Standard-care control group |
| Laura et al. | 2020 | Prospective cohort study | Open esophagectomy | Intervention 63  Control 21 | UK | EC | NAC+surgery | Exercise + Nutrition + Psychological support | 111 | NR | NR | I-IV | 72 | 39 | 68 (61-73) | 67 (62-74) | 8 | From completion of staging investigations to surgery The exact prehabilitation duration variable in different patients | PREPARE program mainly including: Exercise: According to the WHO guidelines, patients were prescribed a personalized exercise program with a defined frequency, intensity, and durationby a experienced exercise therapist Nutritional support: A specialist nutritionist evaluated the nutritional status of patients. Personalised diet plan was designed based on physical condition of each patients Psychological support: Psychotherapies were applied to control anxiety, facilitate adaptation to patients' current psychological health and disease state | Standard-care control group |
| Yuji et al. | 2020 | Retrospective cohort study | MIE + Open esophagectomy | Overall | Japan | ESCC | NAC+surgery | Exercise | 48 | 38 | 10 | I-IV | 23 | 25 | 65.9 ± 7.7 | 65.6 ± 8.7 | 7 | Preoperative 7 days | IMT and self-training such as walking and squat about 1month before surgery combined with aerobic exercise and muscle strength training 7days before surgery | IMT and self-training such as walking and squat about 1month before surgery |
| Christensen et al. | 2018 | Prospective cohort study | MIE-IL + RAMIE + Open esophagectomy | Intervention 20 Control 26 | Denmark | GOJ | NCR+surgery | Exercise | 50 | 45 | 5 | I-III | 21 | 29 | Mean:64.8 | | 8 | Mean: 8.7 weeks | Standard care plus twice-weekly high-intensity aerobic exercise and resistance training sessions | Standard-care control group |
| Enrico et al. | 2018 | RCT | MIS + Open surgery | Intervention 20 Control 15 | Canada | EC+GC | NCR+surgery | Exercise + Nutrition support | 51 | 38 | 13 | I-III | 26 | 25 | 67.3 ± 7.4 | 68.0 ± 11.6 | * | Median: 36 days (17-73) | Individualized, home-based exercise training program 4 times per week according to guidelines provided by the American College of Sports Medicine and nutrition support daily protein intake of 1.2 to 1.5 g/kg of ideal body weight (or approximately 20% of total energy requirements) | Standardized perioperative care |
| Yuji et al. | 2017 | Retrospective cohort study | MIE + Open esophagectomy | Intervention 16 Control 11 | Japan | EC | NCR+surgery | Exercise | 52 | 41 | 11 | 0-IV | 31 | 21 | 64.2 ± 8.9 | 64.9 ± 9.1 | 7 | About preoperative 1 month | Pre-operative IMT | Standard-care control group |
| Kazuyoshi et al. | 2016 | Prospective non-randomized trial | MIG + Open gastrectomy | \ | Japan | GC | Surgery | Exercise + Nutrition support | 90 | 52 | 37 | I-IV | 22 | 68 | 75 ± 5 | 72 ± 4 | 8 | About preoperative 3 weeks | Exercise: handgrip training, walking, and resistance training Nutritional support: daily calorie intake of more than 28 kcal/kg ideal body weight (IBW), and a daily intake of protein of 1.2 g/kg IBW, as well as 2.4 g daily oral supplementation with leucine metabolite β-hydroxy-β-methylbutyrate | Standard-care control group |
| Haruhiko et al. | 2014 | Prospective non-randomized trial | MIG + Open gastrectomy | \ | Japan | GC | Surgery | Exercise | 72 | 69 | 3 | I-III | 18 | 54 | 63.1 (51-76) | 66.1 (39-81) | 8 | About preoperative 4 weeks | Aerobic exercise, resistance training, and stretching The aerobic training was performed 3–7 days per week. The strength of the training was set according to the maximal heart rate reserve to rate the level of perceived exertion Resistance training was carried out once or twice per week Stretching was performed before and after the aerobic training | Standard-care control group |
| Daniela et al. | 2012 | Pragmatic non-RCT | MIE + Open esophagectomy | Intervention 30 Control 17 | Netherland | EC | NCR+surgery | Exercise | 78 | 58 | 20 | NR | 39 | 39 | 65.4 ± 7.5 | 66.5 ± 9.6 | 8 | At least 2 weeks prior to surgery | Pre-operative IMT, trained daily, seven times a week | Standard-care control group |

RCS: retrospective cohort studies; PCS: prospective cohort studies; RCT: randomized controlled trial; NCR: neoadjuvant chemoradiotherapy; NAC: neoadjuvant chemotherapy; S: surgery; MIE: minimally invasive esophagectomy; MIE-IL: minimally invasive Ivor-Lewis esophagectomy; RAMIE: robot-assisted minimally invasive esophagectomy; MIS: minimally invasive surgery; MIG: minimally invasive gastrectomy; EC: esophageal cancer; ESCC: esophageal squamous cell cancer; GOJ: gastroesophageal junction cancer; GC: gastric cancer; E: exercise; N: nutritional support; P: physiologic support; IG: intervention group; CG: control group; NOS: Newcastle–Ottawa Quality Assessment Scale. * The detailed results of Cochrane Collaboration’s Risk of Bias of two RCTs included in this meta-analysis were shown in Figure S1.
