# Supplementary material for: Boosting recovery before surgery: The impact of prehabilitation on upper gastrointestinal cancer patients – A quantitative comparative analysis
Source: PLoS One. 2025 Mar 18;20(3):e0315734. doi: 10.1371/journal.pone.0315734 (PMC11918424; doi:10.1371/journal.pone.0315734)
Supplement: S1 File — (DOCX) [file pone.0315734.s003.docx]

**Search Strategy**

**Medline:**

("Prehabilitation" OR "Rehabilitation" OR "Preoperative" OR "Exercise" OR "Physical treatment" OR "Physical therapy" OR "Aerobic" OR "Resistance training" OR "Nutrition" OR "Nutritional support" OR "Psychological support" OR "Muscle training" OR "Inspiratory muscle training" OR "Recovery" )

AND

("Esophageal cancer" OR "Esophageal carcinoma" OR "Esophageal neoplasm" OR "Oesophageal cancer" OR "Oesophageal neoplasm" OR "Esophageal squamous cell carcinoma" OR "Esophageal adenocarcinoma" OR "Esophageal malignancy" OR "Gastroesophageal junction cancer" OR "Gastroesophageal junction carcinoma" OR "Gastroesophageal junction neoplasms" OR "Esophagogastric junction cancer" OR " Esophagogastric junction carcinoma" OR "Gastric esophageal junction cancer" OR "GEJ cancer" OR "Gastric cancer" OR "Stomach cancer" OR "Gastric carcinoma" OR "Stomach carcinoma" OR "Gastric neoplasms" OR "Upper gastrointestinal tract cancer" OR "Upper GI tract cancer" OR "Upper digestive tract cancer" OR "Upper gastrointestinal neoplasms" )

**Embase:**

('Prehabilitation' OR 'Rehabilitation' OR 'Preoperative' OR 'Exercise' OR 'Physical treatment' OR 'Physical therapy' OR 'Aerobic' OR 'Resistance training' OR 'Nutrition' OR 'Nutritional support' OR 'Psychological support' OR 'Muscle training' OR 'Inspiratory muscle training' OR 'Recovery')

AND

('Esophageal cancer' OR 'Esophageal carcinoma' OR 'Esophageal neoplasm' OR 'Oesophageal cancer' OR 'Oesophageal neoplasm' OR 'Esophageal squamous cell carcinoma' OR 'Esophageal adenocarcinoma' OR 'Esophageal malignancy' OR 'Gastroesophageal junction cancer' OR 'Gastroesophageal junction carcinoma' OR 'Gastroesophageal junction neoplasms' OR 'Esophagogastric junction cancer' OR 'Esophagogastric junction carcinoma' OR 'Gastric esophageal junction cancer' OR 'GEJ cancer' OR 'Gastric cancer' OR 'Stomach cancer' OR 'Gastric carcinoma' OR 'Stomach carcinoma' OR 'Gastric neoplasms' OR 'Upper gastrointestinal tract cancer' OR 'Upper GI tract cancer' OR 'Upper digestive tract cancer' OR 'Upper gastrointestinal neoplasms')

**Cochrane Library:**

("Prehabilitation" OR "Rehabilitation" OR "Preoperative" OR "Exercise" OR "Physical treatment" OR "Physical therapy" OR "Aerobic" OR "Resistance training" OR "Nutrition" OR "Nutritional support" OR "Psychological support" OR "Muscle training" OR "Inspiratory muscle training" OR "Recovery")

AND

("Esophageal cancer" OR "Esophageal carcinoma" OR "Esophageal neoplasm" OR "Oesophageal cancer" OR "Oesophageal neoplasm" OR "Esophageal squamous cell carcinoma" OR "Esophageal adenocarcinoma" OR "Esophageal malignancy" OR "Gastroesophageal junction cancer" OR "Gastroesophageal junction carcinoma" OR "Gastroesophageal junction neoplasms" OR "Esophagogastric junction cancer" OR " Esophagogastric junction carcinoma" OR "Gastric esophageal junction cancer" OR "GEJ cancer" OR "Gastric cancer" OR "Stomach cancer" OR "Gastric carcinoma" OR "Stomach carcinoma" OR "Gastric neoplasms" OR "Upper gastrointestinal tract cancer" OR "Upper GI tract cancer" OR "Upper digestive tract cancer" OR "Upper gastrointestinal neoplasms")

**Web of Science:**

TS=("Prehabilitation" OR "Rehabilitation" OR "Preoperative" OR "Exercise" OR "Physical treatment" OR "Physical therapy" OR "Aerobic" OR "Resistance training" OR "Nutrition" OR "Nutritional support" OR "Psychological support" OR "Muscle training" OR "Inspiratory muscle training" OR "Recovery")

AND

TS=("Esophageal cancer" OR "Esophageal carcinoma" OR "Esophageal neoplasm" OR "Oesophageal cancer" OR "Oesophageal neoplasm" OR "Esophageal squamous cell carcinoma" OR "Esophageal adenocarcinoma" OR "Esophageal malignancy" OR "Gastroesophageal junction cancer" OR "Gastroesophageal junction carcinoma" OR "Gastroesophageal junction neoplasms" OR "Esophagogastric junction cancer" OR "Esophagogastric junction carcinoma" OR "Gastric esophageal junction cancer" OR "GEJ cancer" OR "Gastric cancer" OR "Stomach cancer" OR "Gastric carcinoma" OR "Stomach carcinoma" OR "Gastric neoplasms" OR "Upper gastrointestinal tract cancer" OR "Upper GI tract cancer" OR "Upper digestive tract cancer" OR "Upper gastrointestinal neoplasms")
